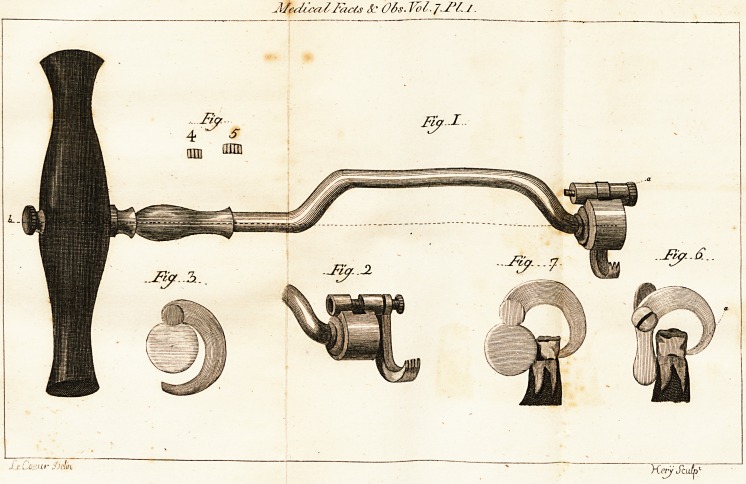# Description of a New Key Instrument for the Extraction of Teeth

**Published:** 1797

**Authors:** J. Savigny

**Affiliations:** Surgical-Instrument Maker in London.


IX. *Deſcription of a new Key Inſtrument for the Extraction of Teeth. Communicated in a Letter to Dr.* Simmons, *by Mr.* J. Savigny, *Surgical-Inſtrument Maker in London.*

*To Dr.* SIMMONS.

SIR,

I BEG leave to preſent you an engraving of a Key Inſtrument, for extracting teeth, which I have lately invented, and in the conſtruction of which I have endeavoured, as much as poſſible, to combine ſimplicity with utility. The complaints I have been continually accuſtomed to hear of the pain and danger attending this operation, have long excited my attention, and have led me to conclude that ſomewhat eſſentially eſſentially deficient muſt have attended the conſtruction of the different inſtruments hitherto employed to effect it.

On comparing and reflecting on the various forms which the key inſtrument has received, I have ever found their principal defect to ariſe from the depth of the bolſter, which, even in the ſmalleſt, deſcribes in its action ſo large a circle (the ſtem or ſhank of the inſtrument being conſidered as its centre) as to occaſion unavoidable inconvenience; and in the larger or deeper ones certain danger of fracturing the alveolar proceſs, and of being followed by conſequences always painful, and frequently dangerous.

The violent effects of ſo powerful a fulcrum have uſually been increaſed by a curve or neck at the inferior extremity of the inſtrument, for the purpoſe of retaining its action in a right line with its handle, when employed in the extraction of the molares in the internal direction; an alteration which, although it effected its intended purpoſe, ſtill augmented the inconveniences I have ſtated to ſo great a degree, that I believe I may ſafely aſſert, it has uniformly produced, more or leſs, the pernicious conſequences mentioned above.

For an eſſential improvement in this part of the the conſtruction of my inſtrument, I acknowledge myſelf indebted to a very intelligent and ingenious paper by Mr. Robert Clarke, of Sunderland, inſerted in your laſt volume *. I found it admirably calculated to aſſiſt the mechanical intention of the circular bolſter I have adopted, the action of which being confined to a revolution upon its own axis, gives ſufficient power for the extraction of the tooth, in nearly a perpendicular direction. A bolſter of this ſhape may be applied without violence to the proceſs, and by preſenting a regular obtuſe ſurface to the gum, leſſens the danger of bruiſing or laceration; while it affords, at the ſame time, a reſiſting point to the claw, in whatever poſition it may be engaged.

The conſtruction of this inſtrument will be beſt underſtood from the annexed engraving †, in which,

Fig. 1. repreſents the inſtrument of its proper ſize and figure; the dotted line is intended to ſhow, that notwithſtanding its advantageous curve, its action is perfectly central, and in a right line with its handle.

* Vol. VI. p. 120.

† See Plate I.

*a* refers

Le Coeur Delin

Hery Sculp^t

*a* refers to a ſcrew with a milled head, retaining the claw :

*b* to another ſcrew with a milled head, ſecuring the handle, which, by this means, is eaſily wholly removed, to render the inſtrument more portable, or its poſition changed, (horizontally or vertically) as may be occaſionally required.

Fig. 2. ſhows the bolſter of the inſtrument, with the claw attached to the projecting extremity, for more conveniently fixing it on the dentes ſapientiæ.

Fig. 3. exhibits a front view of the bolſter, to ſhow its form and circumference.

Fig. 4. and 5. ſhow the dimenſions of the points of the claws, the only difference of ſize requiſite in this inſtrument.

Fig. 6. repreſents the bolſter and curved neck of the common inſtrument applied to a tooth. The dotted line, marked *a*, pointing out the direction of its action, renders any comment upon the conſequences unneceſſary.

Fig. 7. ſhows the bolſter and claw of the improved inſtrument alſo applied to a tooth, by which the comparative difference of the powers of the two inſtruments may be eaſily aſcertained.

The teſt of actual experiment having confirmed the advantages of this inſtrument, I ſubmit this account of it to your conſideration, and if thought worthy a place in your truly important and uſeful publication, its inſertion will be deemed a favour conferred on,

SIR,

Your moſt obedient

And very humble ſervant,

JOHN SAVIGNY.

*King Street, Covent Garden,*

*May* 23, 1796.

X. *Some*

## Figures and Tables

**Fig 1 Fig 2 Fig 3 Fig 4 5 Fig 6 Fig 7 f1:**